# Outcomes of Upper Gastrointestinal Bleeding in Hospitalized Patients With Generalized Anxiety Disorder

**DOI:** 10.7759/cureus.25059

**Published:** 2022-05-16

**Authors:** Alexander J Kaye, Brooke Baker, Sarah Meyers, Sushil Ahlawat

**Affiliations:** 1 Internal Medicine, Rutgers University New Jersey Medical School, Newark, USA; 2 Psychiatry, Rutgers Robert Wood Johnson Medical School, Piscataway, USA; 3 Gastroenterology and Hepatology, Rutgers University New Jersey Medical School, Newark, USA

**Keywords:** upper gastrointestinal bleed, inpatient mortality, selective serotonin reuptake inhibitor, acute renal failure, generalized anxiety disorder (gad)

## Abstract

Background

Upper gastrointestinal bleeding (UGIB) has a high morbidity and mortality. Social deprivation is a risk factor for UGIB and is associated with anxiety. The primary pharmaceutical therapeutic agents for anxiety are selective serotonin reuptake inhibitors. Anxiety is prevalent in the general population and generalized anxiety disorder (GAD) is a common form of anxiety. This study explores the impact of GAD on the outcomes of adult patients hospitalized with UGIB.

Methods

Adult UGIB patients were selected utilizing the National (Nationwide) Inpatient Sample database from year 2014 and International Classification of Diseases, Ninth Revision, Clinical Modification (ICD-9-CM) codes. The outcomes of UGIB patients with and without GAD were investigated. The outcomes explored include inpatient mortality, hypotension/shock, acute renal failure, acute hepatic failure, acute respiratory failure and acute myocardial infarction. A multivariate logistic regression analysis was used to determine if GAD is an independent predictor of the outcomes.

Results

Among 19,850 UGIB patients studied, 2357 had comorbid GAD. GAD was identified as a risk factor for acute renal failure (adjusted odds ratio [aOR] 1.37, 95% confidence interval [CI] 1.30-1.57, p < 0.05) and inpatient mortality (aOR 1.50, 95% CI 1.01-2.06, p < 0.05). The aORs of hypotension/shock, acute hepatic failure, acute respiratory failure and acute myocardial infarction were not statistically significant.

Conclusion

UGIB patients with comorbid GAD are at elevated risk of inpatient mortality and acute renal failure. These results may gain increasing relevance as GAD prevalence has increased since the start of the coronavirus disease 2019 (COVID-19) pandemic.

## Introduction

Upper gastrointestinal bleeding (UGIB) is defined as blood loss originating from the intraluminal gastrointestinal tract proximal to the ligament of Treitz. The three most common etiologies of UGIB are esophageal varices, peptic ulcer disease, and erosive esophagitis [1]. Modifiable risk factors for UGIB include *Helicobacter pylori *infection, alcohol consumption, smoking, peptic ulcer disease, and certain medications including steroids, selective serotonin reuptake inhibitors (SSRIs), nonsteroidal anti-inflammatory drugs (NSAIDs), anticoagulants, aspirin and other anti-platelet agents [2,3]. The non-modifiable risk factors for UGIB include an age of 50 years and older, being male, and having a history of prior UGIB [1,2].

In the United States, UGIB accounts for approximately 300,000 admissions and $2.5 billion dollars in healthcare expenditures per year [4]. The significant hospital resources allocated to UGIB are likely a result of the elevated morbidly and mortality associated with this diagnosis [5,6]. Patients with UGIB are at risk of developing complications such as hypovolemia/shock, acute renal failure and acute respiratory failure [7]. The 30-day mortality of patients who experience UGIB range between 9% and 14% [5].

One notable risk factor for a higher incidence of UGIB is social deprivation [8]. Social deprivation has been described as a "persisting lack of minimally adequate opportunities for decent or supportive human contact including interpersonal interaction, associative inclusion, and interdependent care" [9]. There is an established association between social deprivation, and depressive and anxiety symptoms [10]. Anxiety disorders are common in the general population with about 34% of all individuals experiencing at least one form of anxiety disorder during their lifetime [11]. Generalized anxiety disorder (GAD) is a frequent form of anxiety with a respective lifetime prevalence of 4.6% and 7.7% in male and female patients aged 18-64 years old, respectively [12]. Of note, the prevalence of GAD has been increasing since the onset of the coronavirus disease 2019 (COVID-19) pandemic [13]. The primary pharmacologic treatment of GAD is SSRIs. While social deprivation is associated with both anxiety symptoms and an increased incidence of UGIB, there has been no major study exploring the direct impact anxiety has on the morbidly and mortality of UGIB. This study investigated the outcomes of patients hospitalized with UGIB who also had comorbid GAD. An abstract of this study was accepted to the Digestive Disease Week Annual Meeting, May 2022.

## Materials and methods

This was a retrospective cohort study of all adult patients (patients aged 18 years and older) that were hospitalized for UGIB in year 2014. The data were obtained from the National (Nationwide) Inpatient Sample (NIS), Healthcare Cost and Utilization Project (HCUP), Agency for Healthcare Research and Quality, which is generally recognized as the largest all-payer inpatient database in the United States [14]. The International Classification of Diseases, Ninth Revision, Clinical Modification (ICD-9-CM) codes were used to identify all of the diagnoses and outcomes using the NIS database. The UGIB patients were stratified into one of the two subgroups: those with a history of GAD and those who lacked a history of GAD. Demographic information and hospitalization data including age, sex, race, length of stay, and hospitalization cost were extracted and subsequently compared between the two subgroups. The Charlson Comorbidity Index, a standardized tool used to adjust for confounding variables, was also compared between these subgroups [15,16].

IBM SPSS Statistics, version 28.0 (IBM Corp., Armonk, NY) was used for all of the statistical analyses. The outcomes of interest for this study were hypotension/shock, acute myocardial infarction, acute hepatic failure, acute renal failure, acute respiratory failure, and inpatient mortality. The outcomes for these two groups were then compared. Chi-squared tests and independent t-tests were used to compare the proportions and means, respectively. The statistical analyses performed were two-tailed, and a p-value threshold of under 0.05 was considered statistically significant. Categorical variables were reported as percentages (%) and numbers (N), and continuous variables were reported as means ± standard deviations (SDs). A multivariate logistic regression analysis was also completed to establish if GAD was an independent predictor for the aforementioned clinical outcomes, after adjusting for age, sex race, and Charlson Comorbidity Index.

## Results

A total of 19,850 adult patients hospitalized with UGIB were identified in year 2014. Among these patients admitted with UGIB, 2357 also had a history of GAD. As displayed in Table 1, UGIB patients with GAD were younger (55.4 years old vs. 60.7 years old, p < 0.05), less likely to be female (40.3% vs. 45.5%, p < 0.05), more likely to be Caucasian (78.2% vs. 65.1%, p < 0.05), had a lower Charlson Comorbidity Index (3.04 vs. 3.83, p < 0.05), a shorter length of hospital stay (4.7 days vs. 5.0 days, p < 0.05), and with a less expensive hospitalization ($40,430 vs. $44,610, p < 0.05).

**Table 1:** Demographics, characteristics, length of stay, total hospital charge, and inpatient mortality among upper gastrointestinal bleed patients with and without a history of generalized anxiety disorder

	With generalized anxiety disorder	Without generalized anxiety disorder	p-value
N = 19,850	N = 2,357	N = 17,493	
Patient age, mean (SD)	55.42 (18.81)	60.68 (20.82)	<0.05
Sex, N (%)			<0.05
Female	1,245 (40.3%)	7,957 (45.5%)	
Male	1,406 (59.7%)	9,536 (54.5%)	
Race, N (%)			<0.05
White	1,742 (78.2%)	10,844 (65.1%)	
Black	252 (11.3%)	3,167 (19.0%)	
Hispanic	162 (7.3%)	1,750 (10.5%)	
Asian or Pacific Islander	15 (0.7%)	295 (1.8%)	
Native American	17 (0.8%)	175 (1.1%)	
Other	40 (1.8%)	435 (2.6%)	
Length of stay, in days (SD)	4.70 (4.82)	4.98 (5.92)	<0.05
Total hospital charges, in $ (SD)	40,430 (61,419.41)	44,610 (66,525.90)	<0.05
Charlson Comorbidity Index (SD)	3.04 (2.63)	3.83 (2.79)	<0.05

In Table 2, a comparison of the clinical outcomes in UGIB patients with and without a history of GAD is shown. UGIB patients with a history of GAD had an increased likelihood of acute respiratory failure (5.2% vs. 3.7%, p < 0.05) and patients without a history of GAD had elevated rates of inpatient mortality (3.6% vs. 2.0% , p < 0.05), acute renal failure (18.5% vs. 12.0%, p < 0.05), and acute myocardial infarction (2.8% vs. 1.8%, p < 0.05). No statistically significant difference in hypotension/shock (p = 0.73) and acute hepatic failure (p = 0.83) was found between UGIB patients with and without a history of GAD.

**Table 2:** Unadjusted clinical outcomes among upper gastrointestinal bleed patients with and without a history of generalized anxiety disorder

Outcomes	With generalized anxiety disorder	Without generalized anxiety disorder	p-value
Inpatient mortality	46 (2.0%)	631 (3.6%)	<0.05
Hypotension/shock	264 (11.2%)	2,001 (11.4%)	0.73
Acute renal fail	283 (12.0%)	3,286 (18.5%)	<0.05
Acute hepatic failure	12 (0.5%)	95 (0.5%)	0.83
Acute respiratory failure	88 (5.2%)	903 (3.7%)	<0.05
Acute myocardial infarction	42 (1.8%)	496 (2.8%)	<0.05

The adjusted odds ratios (aOR) of the different clinical outcomes, after adjusting for age, sex race and Charlson Comorbidity Index, are displayed in Table 3. GAD was subsequently identified as an independent risk factor for acute renal failure (aOR 1.37, 95% confidence interval [CI] 1.30-1.57, p < 0.05) and inpatient mortality (aOR 1.50, 95% CI 1.01-2.06, p < 0.05). The p-values for the aORs of acute respiratory failure (aOR 1.25, 95% CI 0.99-1.57, p = 0.06), hypotension/shock (aOR 0.957, 95% CI 0.83-1.10, p=0.54), acute liver failure (aOR 1.25, 95% CI 0.66-2.35, p = 0.50), and acute myocardial infarction (aOR 1.26, 95% CI 0.90-1.76, p = 0.17) did meet the cutoff for statistical significance.

**Table 3:** Multivariate logistic regression analysis of clinical outcomes among upper gastrointestinal bleed patients with and without a history of generalized anxiety disorder *Adjusted for age, sex, race, and the Charlson comorbidity index.

Outcomes	Adjusted odds ratio*	95% Confidence interval	p-value
Inpatient mortality	1.5	1.10-2.06	<0.05
Hypotension/shock	0.96	0.83-1.10	0.54
Acute renal fail	1.37	1.20-1.57	<0.05
Acute hepatic failure	1.25	0.66-2.35	0.50
Acute respiratory failure	1.25	0.99-1.57	0.06
Acute myocardial infarction	1.26	0.90-1.76	0.17

While Table 2 and Table 3 outline the same outcomes, the data appear to be in conflict. For example, in Table 2, acute renal failure and inpatient mortality are seen to be less frequent in the GAD group. In comparison, Table 3 demonstrates these same outcomes occurring more commonly in the GAD group. These differences are due to confounding factors, which are adjusted for in Table 3.

## Discussion

To the best of our knowledge, this is the first study to evaluate the impact of comorbid GAD in patients admitted for UGIB. This study demonstrated that patients hospitalized with UGIB who had comorbid GAD had an increased rate of inpatient mortality and acute renal failure compared to those without GAD. Both of these results can be explained by more difficult to control or increased blood loss [7,17]. One possible explanation for these outcomes may be the use of SSRIs in patients with GAD. An increased risk of bleeding may originate from the anti-platelet effect of SSRIs. SSRIs inhibit the serotonin transport protein, blocking the uptake of serotonin by platelets, which depletes platelet storage of serotonin [18]. Due to decreased storage, less serotonin is released in the setting of vascular injury, diminishing vasoconstriction and platelet aggregation [18]. SSRIs are associated with an elevated risk of UGIB with an odds ratio between 1.9 and 3.6 depending on the dose of the SSRI [19,20]. If a patient is taking an SSRI in combination with NSAIDs or low-dose aspirin, the odds ratio for UGIB increases as high as 12.2 and 5.2, respectively [20].

In addition to SSRIs, the diagnosis of GAD increases the risk of inpatient mortality in UGIB patients. In prior studies, GAD has been found to increase mortality in particular subgroups of patients such as those with cardiovascular disease, breast cancer, as well as those taking oral anticoagulation therapy [21-23]. Two studies found that patients with comorbid GAD had elevated all-cause mortality [24,25]. The pathophysiology for GAD as a risk factor for elevated all-cause mortality remains unknown. In the case of this UGIB sub-population, anxiety may acutely elevate blood pressure and elevated blood pressure has the potential to cause more brisk bleeding [26,27]. Therefore, it is possible this may contribute to GAD’s association with increased mortality and acute renal failure among UGIB patients.

On initial evaluation, the demographic data in Table 1 may appear to be incongruent with the results of Table 3. Table 3 illustrates that UGIB patients with GAD have a higher risk of acute renal failure and inpatient mortality. In contrast, the demographic data in Table 1 of UGIB patients with GAD show that this subgroup had fewer comorbidities, shorter lengths of stay, and lower hospital expenses. However, the GAD group was also found to be younger, which may explain these differences in demographics. In prior studies, younger patients were found to have shorter hospital stays, and fewer comorbidities [28,29]. The shorter hospital stays and fewer comorbidities likely explain the lower hospitalization costs for this group.

There were a few important limitations of this study. One of the key limitations of this study is related to the functionality of performing NIS database research. Performing research with the NIS database depends on the accuracy of billing codes input by healthcare providers, which have the potential to lack precision. Subsequently, inaccurate or imprecise billing codes could have led to under or overrepresentation of the GAD subgroup of patients with UGIB, as well as the outcomes of this study. Another important limitation was the inability to assess for SSRI use due to a lack of an ICD-9 code for SSRI use. Finally, another limitation of this study is that it only captured patients admitted for UGIB. If patients were treated in the emergency department and then discharged with a close outpatient follow-up, the outcomes of these patients were not reflected within this study. Prior research suggests that around 7% of UGIB patients are discharged from the emergency department as a result of being considered low risk for morbidity and mortality due to having low Glasgow-Blatchford Bleeding and Rockall scores [30]. Despite the limitations, a noteworthy strength of this study is its ability to assess patient demographics and outcomes on a national scale. Another strength of the study is the use of a multivariate logistic regression analysis that adjusted for many potential confounding factors.

## Conclusions

In summary, inpatients with GAD who develop UGIB are at an increased risk of mortality and acute renal failure. Because UGIB patients who present with comorbid GAD have an increased mortality risk, enhanced vigilance for early signs of potential complications is warranted. In addition, in the setting of the emergency department, UGIB patients who also are diagnosed with GAD may warrant a lower threshold for admission in light of the higher risk of renal failure and mortality. The findings of this study will likely become increasingly relevant given the high and growing prevalence of GAD. Additional research is necessary to further investigate GAD as an independent risk factor for worse outcomes in the setting of UGIB after controlling for SSRI use.
